# Supplementary material for: Hepatitis C Core Protein Induces a Genotype-Specific Susceptibility of Hepatocytes to TNF-Induced Death In Vitro and In Vivo
Source: Viruses. 2022 Nov 14;14(11):2521. doi: 10.3390/v14112521 (PMC9692671; doi:10.3390/v14112521)
Supplement: Supplementary file 1 [file viruses-14-02521-s001.zip › viruses-1901739 - supplementary.pdf]

Supplementary Figures

```
CLUSTAL O(1.2.4) multiple sequence alignment


core_4a      MSTNPKPQRKTKRNTNRRPMDVKFPGGGQIVGGVYLLPRRGPRLGVRATRKTSERSQPRG    60
core_4f      MSTNPKPQRKTKRNTNRRPMDVKFPGGGQIVGGVYLLPRRGPRLGVRATRKTSERSQPRG    60
             ************************************************************

core_4a      RRQPIPKARRSEGRSWAQPGYPWPLYGNEGCGWAGWLLSPRGSRPSWGPNDPRRRSRNLG    120
core_4f      RRQPIPKARRTEGRSWAQPGYPWPLYGNEGCGWAGWLLSPRGSRPSWGPNDPRRRSRNLG    120
             **********:*************************************************

core_4a      KVIDTLTCGFADLMGYIPLVGAPVGGVARALAHGVRAVEDGINYATGNLPGCSFSIFLLA    180
core_4f      KVIDTLTCGFADLMGYIPLVGAPVGGVARALAHGVRAVEDGINYATGNLPGCSFSIFLLA    180
             ************************************************************

core_4a      LLSCLTVPAST    191
core_4f      LLSCLTVPASA    191
             **********:
```

Figure S1. Clustal Omega alignment of HCV core proteins from the two subtype 4a and 4f strains used in the present study. Bold letters note the amino acid differences between the two strains.

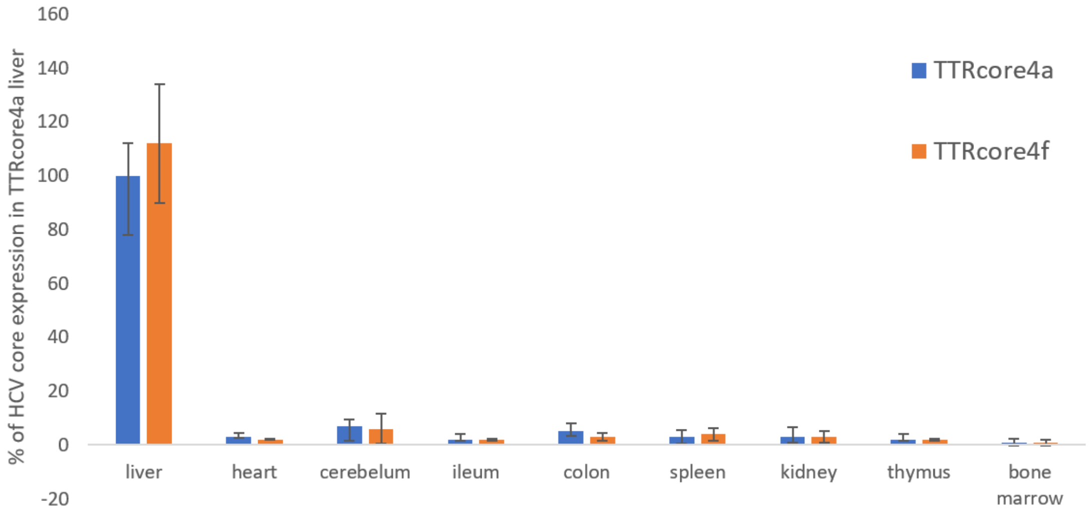

Figure S2. HCV core mRNA expression in various tissues for the assessment of tissue specificity of the expression. Real time RT-PCR and ΔΔCt using GAPDH as normalizer was used to estimate relative expression of HCV mRNA in tissues of the TTRcore4a and TTRcore4f transgenic mice.

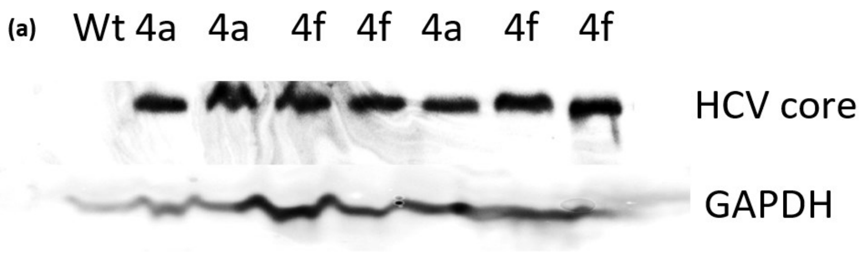

(b)

	Wt	TTRcore4a	ratio
♀	78	69	~1:1
♂	80	76	~1:1
ratio	~1:1	~1:1	

	Wt	TTRcore4f	ratio
♀	59	63	~1:1
♂	65	58	~1:1
ratio	~1:1	~1:1	

Figure S3. (**a**) Western blot of total protein from liver biopsies of TTRcore4a and TTRcore4f mice and a wt control from the first litters of both lines. (**b**) Mendelian ratios of offspring from 50 litters for both TTRcore4a and TTRcore4f lines.

Supplementary Table

Table S1. Oligonucleotide primers for real time RT-PCR used for the verification of the differentially expressed genes in transgenic mice.

Gene Symbol	Forward Primer	Reverse Primer
ADAMDEC1	CCTGGGACTTCTCGGCTAC	TTCGTGAGGCTTTAACTCGGG
SAA1	AAGCTAACTGGAAAAACTCA	TCAGCCATGGTGTCCTCGTGT
SAA1	AAGCTGGCTGGAAAGATGGA	TCAGCCATGGTGTCCTCGTGT
MT1	AAGAGTGAGTTGGGACACCTT	CGAGACAATACAATGGCCTCC
MT2	GCCTGCAAATGCAAACAATGC	AGCTGCACTTGTCGGAAGC
GSN	TCACGGGTGATGCCTATGTCA	AGCCAATAGTGGAGGTCATACTG
HAMP	CTGAGCAGCACCACCTATCTC	TGGCTCTAGGCTATGTTTTGC
HAMP2	CTGAGCAGCACCACCTATCTC	GGCTCTAGGCTCTCTATTCTTCA
GAPDH	AGGTCGGTGTGAACGGATTTG	TGTAGACCATGTAGTTGAGGTCA
CCL2	TTAAAAACCTGGATCGGAACCAA	GCATTAGCTTCAGATTTACGGGT
CCL3	TTCTCTGTACCATGACACTCTGC	CGTGGAATCTTCCGGCTGTAG
CCL5	GCTGCTTTGCCTACCTCTCC	TCGAGTGACAAACACGACTGC
CCL7	GCTGCTTTCAGCATCCAAGTG	CCAGGGACACCGACTACTG
CCL11	GAATCACCAACAACAGATGCAC	ATCCTGGACCCACTTCTTCTT
IL2RG	CTCAGGCAACCAACCTCAC	GCTGGACAACAAATGTCTGGTAG
CFLAR (cFLIP)	GCTCCAGAATGGGCGAAGTAA	ACGGATGTGCGGAGGTAAAAA
